# Expression of parathyroid hormone/parathyroid hormone-related peptide receptor 1 in normal and diseased bladder detrusor muscles: a clinico-pathological study

**DOI:** 10.1186/1471-2490-15-2

**Published:** 2015-01-21

**Authors:** Nobuyuki Nishikawa, Rie Yago, Yuichiro Yamazaki, Hiromitsu Negoro, Mari Suzuki, Masaaki Imamura, Yoshinobu Toda, Kazunari Tanabe, Osamu Ogawa, Akihiro Kanematsu

**Affiliations:** Department of Urology, Kyoto University Graduate School of Medicine, Kyoto, Japan; Department of Cell Physiology, Nagoya City University Graduate School of Medical Sciences, Nagoya, Japan; Department of Urology, Tokyo Women’s Medical University, Tokyo, Japan; Department of Urology, Kanagawa Children’s Medical Centre, Yokohama, Japan; Department of Urology, Otsu Red Cross Hospital, Otsu, Japan; Department of Clinical Laboratory Science, Tenri Health Care University, Tenri, Japan; Department of Urology, Hyogo College of Medicine, Nishinomiya, Japan

**Keywords:** Parathyroid hormone-related peptide, Parathyroid hormone 1 receptor, Bladder compliance, Smooth muscle

## Abstract

**Background:**

To investigate the expression of parathyroid hormone (PTH)/PTH-related peptide (PTHrP) receptor 1 (PTH1R) in clinical specimens of normal and diseased bladders. PTHrP is a unique stretch-induced endogenous detrusor relaxant that functions via PTH1R. We hypothesized that suppression of this axis could be involved in the pathogenesis of bladder disease.

**Methods:**

PTH1R expression in clinical samples was examined by immunohistochemistry. Normal kidney tissue from a patient with renal cancer and bladder specimens from patients undergoing ureteral reimplantation for vesicoureteral reflux or partial cystectomy for urachal cyst were examined as normal control organs. These were compared with 13 diseased bladder specimens from patients undergoing bladder augmentation. The augmentation patients ranged from 8 to 31 years old (median 15 years), including 9 males and 4 females. Seven patients had spinal disorders, 3 had posterior urethral valves and 3 non-neurogenic neurogenic bladders (Hinman syndrome).

**Results:**

Renal tubules, detrusor muscle and blood vessels in normal control bladders stained positive for PTH1R. According to preoperative urodynamic studies of augmentation patients, the median percent bladder capacity compared with the age-standard was 43.6% (range 1.5–86.6%), median intravesical pressure at maximal capacity was 30 cmH_2_O (range 10–107 cmH_2_O), and median compliance was 3.93 ml/cmH_2_O (range 0.05–30.3 ml/cmH_2_O). Detrusor overactivity was observed in five cases (38.5%). All augmented bladders showed negative stainings in PTH1R expression in the detrusor tissue, but positive staining of blood vessels in majority of the cases.

**Conclusions:**

Downregulation of PTH1R may be involved in the pathogenesis of human end-stage bladder disease requiring augmentation.

## Background

Relaxation of the detrusor muscle is a fundamental requirement for normal bladder storage function. Severe failure of this relaxation mechanism causes upper urinary tract deterioration as a result of abnormal elevation of intravesical pressure [1]. Such extreme pathology is termed low-compliance bladder, and is typically seen in paediatric cases with congenital spinal disorders, posterior urethral valves, and also in rare forms of severe non-neurogenic neurogenic bladder (Hinman syndrome). The primary goal in treating such patients is to prevent urinary tract damage by maintaining low-pressure storage and effective bladder evacuation [2]. This is usually achieved through medical therapy using antimuscarinic drugs combined with clean intermittent catheterization. However, if these conservative therapies fail, bladder augmentation using the digestive tract is indicated, though such surgery may lead to various long-term complications, including metabolic acidosis, bowel dysfunction, rupture, and risk of secondary malignancies [3, 4]. However, despite these clinical problems, the molecular mechanisms underlying low-compliance end-stage bladder disease have not yet been thoroughly investigated [4].

Parathyroid hormone-related peptide (PTHrP) was originally identified as a cause of hypercalcemia in paraneoplastic syndrome, [5] and has since been found to be expressed in most systemic tissues, with diverse physiological roles [6, 7]. We previously reported that PTHrP acted as a stretch-induced endogenous relaxant of detrusor muscle [8]. PTHrP functions via the PTH/PTHrP receptor 1 (PTH1R), which is expressed in detrusor muscle but not in urothelium. In rat experiments, PTHrP peptide potently suppressed spontaneous contraction of detrusor muscle strips, and intravenous administration of PTHrP peptide increased bladder compliance [8]. These results suggest that endogenous PTHrP may inhibit the abnormal decrease in bladder compliance by bladder distention. We hypothesized that PTH1R should also be expressed in human bladder detrusor muscle, and that suppression of this axis could be involved in the pathogenesis of end-stage low-compliance bladder.

In this study, we therefore investigated the expression of PTH1R in clinical specimens from patients with normally functioning bladder and those undergoing bladder augmentations, to explore the involvement of the PTHrP-PTH1R axis in normal and diseased bladder detrusor muscle.

## Methods

### Clinical specimens

This study was authorized by the Institutional Review Board of Kyoto University (G279) and Tokyo Women’s Medical University (2089), and written informed consents for participation in the study were obtained from participants or parents. Normal kidney tissue, as a positive control of PTH1R, was obtained from a nephrectomy specimen from a patient with renal cell carcinoma without paraneoplastic hypercalcemia, and was used as a positive control for the staining procedure. Bladder specimens were obtained from the bladder dome by sagittal section in following patients. In total, 3 normal control bladder tissues were obtained during cystotomy for ureteral reimplantation for vesicoureteral reflux in two patients, and partial cystectomy for urachal cyst in one patient, who all underwent surgery at the Department of Urology, Kyoto University Hospital. These three patients had no symptoms of abnormal bladder storage or emptying. The clinical backgrounds of the control patients are summarized in Table 1. Data are expressed as mean ± S.D.

**Table 1:** **Clinical background and immunostaining result of the control tissue**

Sample	Underlying disease	Gender	Age	IHC-PTH1R	
				Detrusor	Blood vessels
Normal Kidney	Renal Cell Carcinoma	M	54	N/A	+
Normal bladder-1	Urachal cyst	F	57	+	+
Normal bladder-2	Vesicoureteral reflux	F	1	+	+
Normal bladder-3	Vesicoureteral reflux	F	12	+	+

IHC, immunohistochemistry.

In total, 13 diseased bladder tissues were obtained from patients who underwent bladder augmentation at the Department of Urology, Tokyo Women’s Medical University. The clinical backgrounds of the patients are summarized in Table 2. The median age of the patients was 15 years (range 8–31 years, mean 17.8 ± 8.8 years), and included 9 males and 4 females. Seven patients had spinal disorders, three had posterior urethral valves and three had Hinman syndrome. All the patients underwent preoperative urodynamic studies combined with cystography. Bladder augmentation was indicated for low-compliance bladder and/or incontinence, despite aggressive anti-cholinergic regimens. The bladder capacity was presented as the percentage of the reported age-standard capacity for Japanese children, calculated by the formula 25 × (age in years + 2) for patients younger than 12 years, and 350 ml was defined as 100% for patients older than 12 years [9].

**Table 2:** **Clinical background and staining results of the diseased bladder**

	Gender	Age	Maximal Bladder Capacity		Intravesical Pressure at Maximal Capacity (cmH _2_O)	Compliance (ml/cmH _2_O)	DO	VUR	Leak Point Pressure (cmH _2_O)	IHC-PTH1R	
			Maximal Capacity (ml)	Percent capacity of age standard						Detrusor	Blood vessels
Myelomeningocele-1	M	14	60	17.1	40	1.5	-	-	Incontinent	-	-
Myelomeningocele-2	F	15	117	33.4	53	2.21	-	+	>60	-	+
Myelomeningocele-3	M	20	250	71.4	24	10.4	-	-	25	-	-
Myelomeningocele-4	F	27	147	42.0	30	4.9	-	+	22.5	-	-
Myelomeningocele-5	F	29	212	60.6	23	9.22	+	+	25	-	-
Sacral agenesis-1	M	30	146	41.7	30	4.87	-	+	NA	-	+
Sacral agenesis-2	F	31	266	76.0	30	8.87	-	+	NA	-	-
Posterior urethral valve-1	M	8	109	43.6	47	2.32	-	-	>40	-	+
Posterior urethral valve-2	M	10	147	49.0	40	3.68	-	+	>40	-	+
Posterior urethral valve-3	M	11	5	1.5	107	0.05	+	-	NA	-	-
Hinman syndrome-1	M	8	118	47.2	30	3.93	+	+	>60	-	+
Hinman syndrome-2	M	10	117	39.0	40	2.93	+	-	Incontinent	-	+
Hinman syndrome-3	M	18	303	86.6	10	30.3	+	+	NA	-	+

DO, detrusor overactivity; VUR, vesicouteral reflux; IHC, immunohistochemistry.

### Immunohistochemistry

The specimens were fixed in formalin and paraffin-embedded. Sections were mounted on glass slides and used for immunohistochemical detection of PTH1R. Incubation and washing procedures were carried out at room temperature, unless otherwise specified. Deparaffinization and antigen retrieval were carried out in a microwave, as described previously, [10] and endogenous peroxidase activity was then blocked with 0.3% H_2_O_2_ in methyl alcohol for 30 min. The glass slides were washed six times in phosphate-buffered saline (PBS) for 5 min each, and mounted with 1% horse normal serum in PBS for 30 min for pre-blocking. Primary antibody against PTH1R (3D1.1, Santa Cruz Biotechnology, Dallas, TX, USA; 1:100) was then applied overnight at 4°C, followed by incubation with biotinylated horse anti-mouse serum (second antibody) (ABC-Elite, Vector Laboratories, Burlingame, CA, USA) diluted 1:300 in PBS for 40 min, followed by six washes in PBS (5 min each). Avidin-biotin-peroxidase complex (ABC-Elite, Vector Laboratories, Burlingame, CA) was applied for 50 min at a dilution of 1:100 in bovine serum albumin. After washing six times in PBS (5 min each), a coloring reaction was carried out using diaminobenzidine, with a uniform reaction time for all specimens. Nuclei were counterstained with haematoxylin. PTH1R protein-expression intensity was classified as negative or positive by two independent examiners, with reference to renal tubules as a positive immunostaining control.

## Results

### Immunostaining study with PTH1R of a kidney and normal control bladders

**Figure 1:**
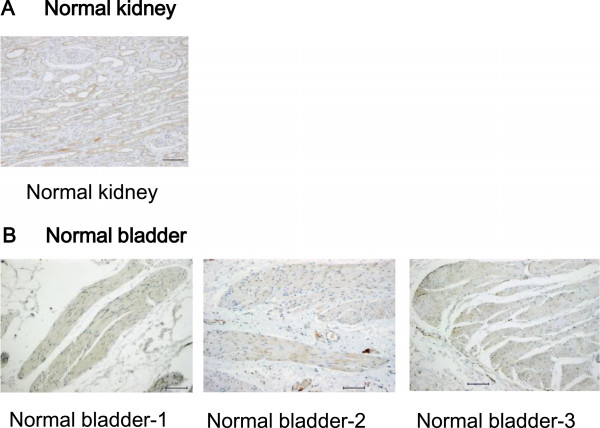
**PTH1R immunohistochemical staining of normal kidney tissue and normal control bladder detrusor tissues. (A)** Renal tubules were positively stained, in contrast to faint staining in glomeruli. **(B)** Cytoplasm of normal bladder detrusor muscle cells and blood vessels showed positive staining with PTH1R antibody. Scale bar: 100 μm.

In the kidney specimen, the tubules stained positive for PTH1R, in contrast to the glomeruli, which stained faint, as reported previously (Figure 1A) [11]. In the normal control bladder specimens, both the detrusor tissue and blood vessel stained positive for PTH1R. Staining in the detrusor tissue was mainly observed in the cytosol (Figure 1B).

### Preoperative urodynamic study

Preoperative urodynamic study revealed that the bladders in all patients undergoing bladder augmentation had deteriorated storage function. The median bladder capacity was 146 ml (range 5–303 ml, mean 153.6 ± 84.0 ml), median percent capacity of age-standard was 43.6% (range 1.5–86.6%, mean 46.9 ± 23.2%), median intravesical pressure at maximal capacity was 30 cmH_2_O (range 10–107 cmH_2_O, mean 38.8 ± 23.3cmH_2_O), and the median compliance was 3.93 ml/cmH_2_O (range 0.05–30.3 ml/cmH_2_O, mean 6.6 ± 7.8 ml/cmH_2_O). Detrusor overactivities were noted in five patients and vesicoureteral reflux in eight patients (Table 2).

### Immunostaining study with PTH1R of low compliance bladder

**Figure 2:**
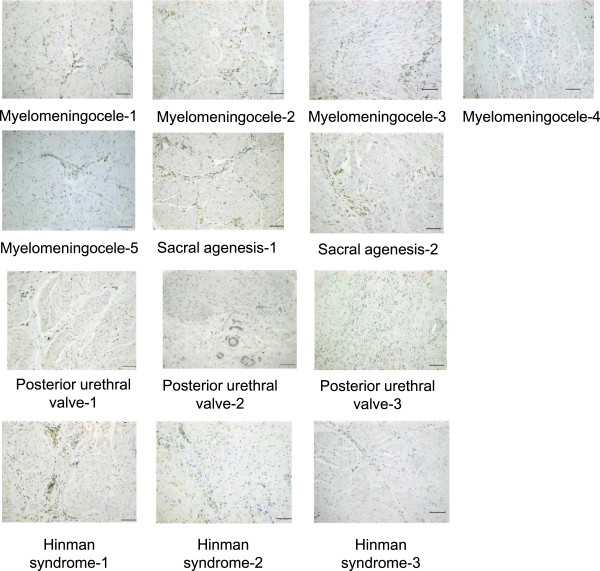
**PTH1R immunohistochemical staining of diseased bladder detrusor tissues.** All diseased bladder detrusor tissues showed negative staining with PTH1R antibody, regardless of the underlying diseases, but positive staining of blood vessels was retained in many specimens. Scale bar: 100 μm.

All of the bladder-augmentation specimens showed negative PTH1R staining in detrusor smooth muscle cells in the bladder wall, in contrast to the positive staining seen in normal control bladder specimen. However, blood vessels stained positive for PTH1R in seven out of 13 augmentation cases (53.8%), indicating that the negative staining was not the result of a technical failure, but rather reflected downregulation of PTH1R expression in these bladders (Figure 2).

## Discussion

This paper demonstrated the expression of PTH1R in normal bladder tissue and its downregulation in end-stage low-compliance bladders requiring augmentation. Combined with the potent relaxant effect of PTHrP peptide in rat bladder reported in our previous study, [8] these findings suggest the functional involvement of this axis in normal human bladder physiology, and its loss in severely diseased bladders with decreased compliance.

The relaxant effect of PTHrP in the bladder has been reported previously [12, 13] Yamamoto et al. focused on stretch-induced upregulation of PTHrP in the bladder in vivo, [12] which effect was replicated in cultured smooth muscle cells under stretch by Steers et al [13]. However, PTHrP showed only modest suppression of carbachol-induced contraction of bladder strips in those studies, leaving the physiological relevance of this observation unanswered. In contrast, our recent study in rats showed a potent relaxant effect of PTHrP in suppressing the spontaneous contraction of detrusor strips, and thus increasing bladder compliance in vivo [8]. Our study also demonstrated that PTH1R was expressed primarily in the muscle layer, and not in the urothelial layer. As a logical extension of this previous study, we investigated the expression of PTH1R in the human bladder, and this report supplements and advances the previous study, by investigating the clinical relevance of this axis utilizing human samples.

Detrusor muscle tissues in normal control bladders stained positive for PTH1R, especially in the cytosol. This result seems to contradict the fact that PTH1R is a membrane-anchored G protein-coupled receptor, [14] allowing binding of PTHrP at the membrane surface. However, PTH1R translocation from the plasma membrane into the cytosol after incubation with the PTH1R agonist, PTH (1–34), and positive staining in the cytosol was also seen in other organs such as acinar cells of the prostate gland, cell clusters within the adrenal cortex and cells of the epithelial hair sheath [11].

In sharp contrast, the detrusor muscle in augmented bladders showed negative staining for PTH1R. This lack of staining did not indicate a technical failure, given that blood vessels stained positively in seven out of 13 (53.8%) cases. The negative staining of vessels in the remaining six cases may have been associated with deterioration of the vessels themselves, or technical failure caused by different fixation conditions. If PTH1R is downregulated in these bladders, they may not be able to respond to endogenous PTHrP, which could function as a protective relaxant against excessive distention. Downregulation of PTH1R is thus consistent with low bladder compliance.

In regard of the functional effect in PTHrP-PTH1R axis in the bladder, not only the receptor PTH1R, but expression level of the ligand PTHrP, in normal and diseased bladder is also of a great interest. Perez-Martinez et al. reported about PTHrP immunostaining in rabbit bladder outlet obstruction model [15]. However, we did not succeed in visualizing PTHrP signal in paraffin-embedded human bladder, nor in rat bladder under various fixation conditions. Therefore we did not focus on expression of PTHrP in this study.

Further physiological experiments would have been ideal to confirm these speculations, demonstrating the unresponsiveness of diseased bladder strips to PTHrP peptide. Similarly, it may be of interest whether the difference in expression level of PTH1R may be attributed to altered production, degradation, release, or removal. Unfortunately, it was practically impossible to obtain materials at the separate time of surgery and subsequently perform physiological experiment or obtain enough materials for various biochemical assays. Such mechanistic part should be better studied in experimental model system such as cultured cells and animals, as we did in our previous report, rather than in clinical samples. Therefore it is inevitable limitation of the present study design that it is unidimensional, but supplemented by our previous mechanistic study, it addresses more direct clinical relevance.

Clinical translation of the results of this study may not be straightforward. If downregulation of PTH1R is a feature of low-compliance bladders, it would be difficult to use this axis as a target for treatment. However, it is possible that bladders still positive for PTH1R may retain the ability to relax against excessive distention, while downregulation of PTH1R could be a marker for unresponsiveness to conservative therapy, indicating the need for bladder augmentation. In addition, the PTHrP-PTH1R axis could be a target for treating milder damage in bladders retaining PTH1R expression, including overactive bladder, which affects a far larger percentage of the population than severely diseased bladder. Unfortunately, currently-available PTH1R agonists have systemic side effects that preclude their use for bladder diseases, and bladder-specific derivatives are awaited to allow the clinical translation of the PTHrP-PTH1R axis for treating bladder diseases.

## Conclusions

In conclusion, we demonstrated PTH1R expression in the smooth muscle in normal bladders, in contrast to negative expression in muscle from severely diseased bladders. Suppression of the PTHrP-PTH1R axis may therefore be involved in bladder pathophysiology.

## Abbreviations

PTH: Parathyroid hormone
PTHrP: Parathyroid hormone-related peptide
PTH1R: Parathyroid hormone/ parathyroid hormone-related peptide receptor 1
PBS: Phosphate-buffered saline.
